# Comparative Proteomic Analysis of Developmental Changes in P-Type Cytoplasmic Male Sterile and Maintainer Anthers in Wheat

**DOI:** 10.3390/ijms22042012

**Published:** 2021-02-18

**Authors:** Yamin Zhang, Qilu Song, Lili Zhang, Zheng Li, Chengshe Wang, Gaisheng Zhang

**Affiliations:** National Yangling Agricultural Biotechnology & Breeding Center, Yangling Branch of State Wheat Improvement Centre, College of Agronomy, Northwest A&F University, Yangling 712100, China; ymzhang2017@163.com (Y.Z.); songqilu1234@163.com (Q.S.); zll3142015@163.com (L.Z.); lizheng9045@126.com (Z.L.)

**Keywords:** wheat, cytoplasmic male sterility, anther proteomics, microspore development

## Abstract

Cytoplasmic male sterility (CMS) plays an important role in the application of heterosis in wheat (*Triticum aestivum* L.). However, the molecular mechanism underlying CMS remains unknown. This study provides a comprehensive morphological and proteomic analysis of the anthers of a P-type CMS wheat line (P) and its maintainer line, Yanshi 9 hao (Y). Cytological observations indicated that the P-type CMS line shows binucleate microspore abortion. In this line, the tapetum degraded early, leading to anther cuticle defects, which could not provide the nutrition needed for microspore development in a timely manner, thus preventing the development of the microspore to the normal binucleate stage. Proteomic analysis revealed novel proteins involved in P-type CMS. Up to 2576 differentially expressed proteins (DEPs) were quantified in all anthers, and these proteins were significantly enriched in oxidative phosphorylation, glycolysis/gluconeogenesis, citrate cycle (TCA cycle), starch and sucrose metabolism, phenylpropanoid biosynthesis, and pyruvate metabolism pathways. These proteins may comprise a network that regulates male sterility in wheat. Based on the function analysis of DEPs involved in the complex network, we concluded that the P-type CMS line may be due to cellular dysfunction caused by disturbed carbohydrate metabolism, inadequate energy supply, and disturbed protein synthesis. These results provide insights into the molecular mechanism underlying male sterility and serve as a valuable resource for researchers in plant biology, in general, and plant sexual reproduction, in particular.

## 1. Introduction

Heterosis is defined as the superiority of the heterozygous hybrid progeny over both homozygous parents. Plant male sterility is a highly valued trait in plant breeding and hybrid production. Plant male sterility is classified into two categories: genic male sterility (GMS) and cytoplasmic CMS [1]. CMS is caused by mitochondrial genes together with nuclear genes, which leads to abnormal anther development and pollen abortion [2,3]. CMS has been identified and characterized in more than 150 plant species, including green bean (*Phaseolus vulgaris* L.), pearl millet (*Pennisetum glaucum* (L.)R.Br.), sugar beet (*Beta vulgaris* L.), carrot (*Daucus carota* L.), maize (*Zea mays* L.), onion (*Allium cepa* L.), petunia (*Petunia hybrida* ‘Mitchell’), rice (*Oryza sativa* L.), rye (*Secale cereale* L.), sunflower (*Helianthus annuus* L.), barley (*Hordeum vulgare* L.), and wheat (*Triticum aestivum* L.), and has been used in commercial hybrid production [4,5]. Studies on heterogeneous male sterile wheat lines began in 1951, when a CMS line was first generated using *Aegilops caudata*. Subsequently, more than 130 nuclear and cytoplasmic hybrids were obtained, which exhibited T-, K-, V-, D-, A-, or P-type CMS. Moreover, within Triticeae, the genus *Aegilops* showed the most successful distant hybridization with wheat [6]. 

Genetically speaking, common wheat (*Triticum aestivum* L.; AABBDD genome) is an allohexaploid species which is thought to have emerged through natural hybridization of three diploid donor species (*Triticum urartu*, AA; *Aegilops speltoides*, SS; and *Aegilops tauschii* Coss, DD) [7,8]. As the population increases, nowadays, wheat has become one of the most important food crops in the world, with the highest area under cultivation worldwide. Heterosis has been employed in wheat to increase grain yield and to produce hybrid lines. In wheat, male reproductive processes occur within the anther. In anthers, diploid sporogenous cells go through meiosis to form haploid microspores, which eventually develop into pollen grains or the male gametophyte [9,10]. Anther development involves many complex processes, and any disorder in anther development can lead to pollen abortion, thus resulting in male sterility [11]. Previous studies on male sterility in wheat have largely focused on changes in gene expression, enzyme activity, and hormone metabolism; however, the cause of wheat anther abortion abortive has not yet been explored at the proteomic level [12,13].

In recent years, proteomic approaches have been used to study anther development and pollen reproduction in many plant species, including *Arabidopsis thaliana* [14], rice [15,16], and tomato (*Solanum lycopersicum* L.) [17]. Anther proteomics analysis has led to the identification of many proteins specifically expressed in anthers, most of which are involved in pollen development, tapetum degradation, programmed cell death (PCD), and callose hydrolyzation [18,19,20,21]. Furthermore, a large number of proteins involved in energy conversion, signal transduction, stress tolerance, transcription, and protein metabolism have also been characterized in pollen [15,17,22]. Because the direct identification of male-sterility-related genes is difficult, researchers examine differences in expression patterns between the male sterile mutant and the corresponding wild-type plant. In tomato, proteomic analysis of male sterile 7B-1 mutant anthers at the tetrad stage revealed that levels of proteasome and 5B protein, associated with tapetum degeneration, were down-regulated, thus indicating their role in male sterility [23]. In *Brassica napus*, proteins associated with carbohydrate and energy metabolism, photosynthesis, and flavonoid synthesis were downregulated in CMS anthers, indicating the roles of these proteins in pollen development [24]. These studies provide important insights into the genetic control of mitochondrial and nuclear interactions in CMS systems. 

Label-free quantitative methods that measure mass spectral peak intensities or number of MS/MS spectra are accurate in quantitating protein abundances [25,26]. Low-abundance proteins can be filtered out when randomly detected in the field of quantitative proteomics. Appropriate statistical methods and expertise have been used to analyze such spectral data. Although isotope-labeling methods are considered more accurate than label-free methods, the latter are considered advantageous and convenient for global protein expression studies because of their greater dynamic range and proteome coverage as well as simpler experimental protocol [27].

**Figure 1:**
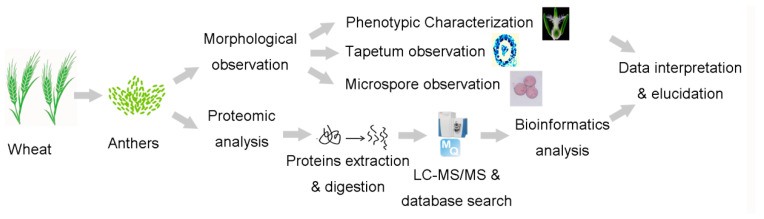
Outline of experimental workflow.

In this study, we aimed to gain further insights into some of the functional mechanisms controlling CMS. Therefore, P (A P-type CMS line of wheat; P) and Y (maintainer line Yanshi 9 hao; Y) anthers at the uninucleate stage (defined stage 2) and the binucleate stage (defined stage 4), respectively, were used by label-free quantitation methods, in combination with other complementary molecular and physiological techniques, to examine protein and gene expression levels. The workflow and experimental design used in this study are outlined in Figure 1.

## 2. Results

### 2.1. Observation of Defects during Anther Development in the CMS Line

**Figure 2:**
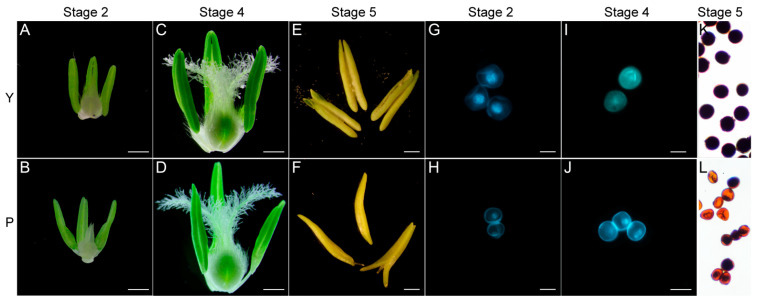
Phenotype of anthers and microspores in different stages in male sterile (P) and its maintainer (Y). Stage 2 (**A**,**B**,**G**,**H**): Early uninucleate stage; Stage 4 (**C**,**D**,**I**,**J**): Binucleate stage; and Stage 5 (**E**,**F**,**K**,**L**): Trinucleate stage. Bars: 500 µm in (**A**–**F**); 50 µm in (**G**–**J**).

To identify differences in anther morphology, P-type CMS line and its maintainer line were compared at stages 2 and 4. No significant difference was detected in anther phenotype at stages Y2 and P2 (Figure 2A,B). At stage 4, anthers of P were thin and shriveled compared with those of the maintainer line (Figure 2C,D). Next, the development of microspores was observed at two stages of Y and P by DAPI and acetocarmine staining. The results showed no significant difference in microspore development at stages Y2 (Figure 2G and Figure S1C) and P2 (Figure 2H and Supplemental Figure S1D). At stage 4, microspores were turgid and round, and the nucleus divided normally into two nuclei in the maintainer line (Figure 2I and Figure S1G). By contrast, microspores of the P-type CMS line were irregular in shape, plasmolysis occurred, and the nucleus failed to divide normally into two nuclei (Figure 2J and Figure S1H). At the mature pollen stage (Figure 2E,F), anthers were not dehiscent and pollen grains were stained brown by 2% I_2_-KI (Figure 2K,L) compared with Y.

### 2.2. Cytological Analysis of Microspore Development

**Figure 3:**
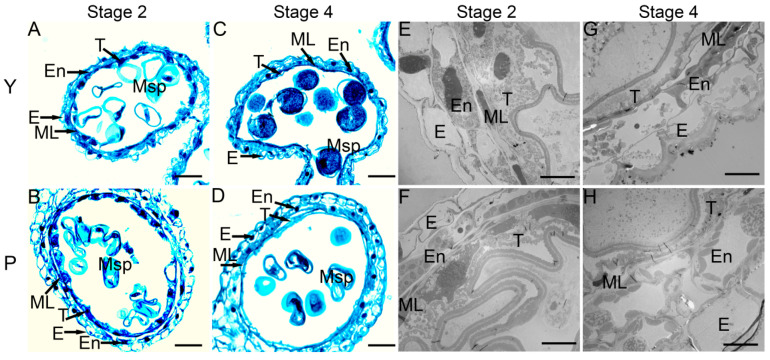
Observation of transverse sections (**A**–**D**) and transmission electron microscope (TEM) (**E**–**H**) of different stages in male sterile line (P) and its maintainer line (Y). (**A**–**D**), stained with safranin O-fast green. Stage 2 (Early uninucleate stage): (**A**,**B**,**E**,**F**); Stage 4 (Binucleate stage): (**C**,**D**,**G**,**H**). E, En, ML, T, and Msp indicate the epidermis, the endothecium, the middle layer, the tapetum, and the microspore, respectively. Bars: 50 µm (**A**–**D**); 10 µm (**E**–**H**).

To further investigate when the defects occurred during microspore development, anthers of the P-type CMS line and its maintainer line were collected, and the transverse sections of anthers were examined by staining with safranin O-fast green. At the tetrad stage, the epidermis, endothecium, middle layer, and tapetum of anthers were clearly visible (Figure S2A,B). The tapetum cells showed a dense cytoplasm and were deeply stained (Figure S2A,B). It is worth noting that some tapetum cells began to shed, and the cytoplasm began to vacuolize, indicating the onset of apoptosis. All four cell layers were found in Y2 and P2 anthers (Figure 3A,B) but with significant differences; in Y2 anthers, the tapetal cells were relatively thick, and the cytoplasm was deeply stained in Y2 plant (Figure 3A), whereas in P2 anthers, the tapetal cells showed weak staining and were relatively narrow (Figure 3B). At stage 4, the tapetum cells were almost completely degenerated, and microspores of the P-type CMS line were shrunken and failed to store enough nutrients for further development compared with those of the maintainer line (Figure 3C,D).

To further confirm the above results, we analyzed the anthers by transmission electron microscopy (TEM). At the tetrad stage, vacuolization of the tapetum was clearly evident, and the tapetum cells were loosely arranged, suggesting early degeneration and later shedding compared with Y anthers (Figure S2C,D). At stage 2, the cytoplasm of tapetum cells in both the CMS line and its maintainer line was condensed toward the center. Compared with Y anthers, the tapetum layers in the P anthers showed more rapid degeneration (Figure 3E,F). At stage 4, unlike Y anthers, the P anthers showed few remnants of tapetum cells (Figure 3G,H). These results are in complete agreement with the above observations.

### 2.3. Proteomic Analysis of Anthers at Different Stages

We performed proteomics analysis of P and Y anthers at two developmental stages (stage 2 and stage 4) using the label-free quantitation method. A total of 400,169 spectra were generated. Of these, 166,656 spectra, representing 21,694 identified peptides and 13,010 specific peptides, were matched. Ultimately, 4519 proteins were identified, and 2576 proteins were quantified in all anthers (Table S1), and the latter were used for subsequent analysis. Most of the identified peptides contained 7–22 amino acid residues (Figure S3A). Of the 4519 proteins, 3766 (83.34%) proteins contained at least two unique peptides, while 753 (16.66%) proteins contained only one unique peptide (Figure S3B). All identified proteins could be divided into four molecular weight classes: <10 kDa (101 proteins; 2.24%), 10–70 kDa (3622; 80.15%), 70–120 kDa (660; 14.61%), and >120 kDa (136; 3.01%) (Figure S3C). Additionally, the protein sequence coverage distribution showed that the sequence coverage decreased with the increase in protein molecular weight, and the amount of protein decreased with the increase in protein sequence coverage (Figure S3D,E).

### 2.4. Analysis of DEPs during Anther Development

**Figure 4:**
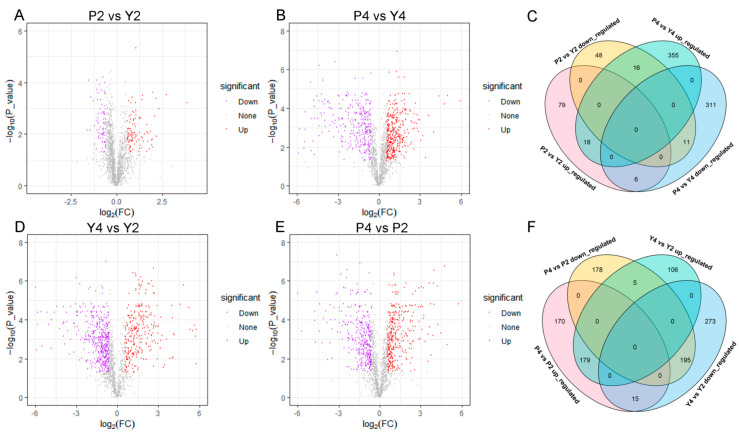
Analysis of the DEPs in four comparison groups, respectively. (**A**,**B**,**D**,**E**) Volcano plots of the DEPs in four comparison groups; (**C**) Venn diagrams of upregulated and downregulated DEPs between P2 vs. Y2 and P4 vs. Y4; (**F**) Venn diagrams of upregulated and downregulated DEPs between Y4 vs. Y2 and P4 vs. P2.

To identify proteins associated with CMS in wheat, we compared protein expression in stage 2 (P2 vs. Y2) and 4 (P4 vs. Y4). A total of 178 and 717 DEPs (fold change > 1.5; *p* < 0.05) were identified (Figure 4A,B, Table S2), of which 103 proteins were upregulated and 75 were downregulated at stage 2, while 389 proteins were upregulated and 328 were downregulated at stage 4 (Figure S4A). The number of upregulated DEPs was 28 and 61 more than that of downregulated DEPs in stage 2 and 4, respectively, whereas the number of upregulated and downregulated DEPs in stage 4 was 286 and 253 more than those in stage 2, respectively (Figure S4A). A total of 51 DEPs were common to both stages (Figure 4C, Table S3), of which 18 were upregulated, 11 were downregulated, 6 were initially upregulated and then downregulated, and 16 were initially downregulated and then upregulated.

To observe changes in protein expression within a genotype during anther development, we performed Y4 vs. Y2 and P4 vs. P2 comparisons and obtained 773 (290 upregulated and 483 downregulated) and 742 DEPs (364 upregulated and 378 downregulated), respectively (Figure 4D,E, Table S2, Figure S4B). These data show that the number of downregulated DEPs was 193 more than that of upregulated DEPs in the maintainer line. Meanwhile, the number of upregulated and downregulated DEPs was almost the same in sterile lines (Figure S4B). Additionally, 394 DEPs were common to both comparison groups (Y4 vs. Y2; P4 vs. P2) (Figure 4F, Table S3). Among them, 179 were upregulated and 195 were downregulated in both comparison groups; 5 were upregulated in comparison group Y4 vs. Y2 and downregulated in comparison group P4 vs. P2; 15 were downregulated in comparison group Y4 vs. Y2 and upregulated in comparison group P4 vs. P2. 

### 2.5. Subcellular Localization Prediction and Domain Enrichment Analysis of DEPs

**Figure 5:**
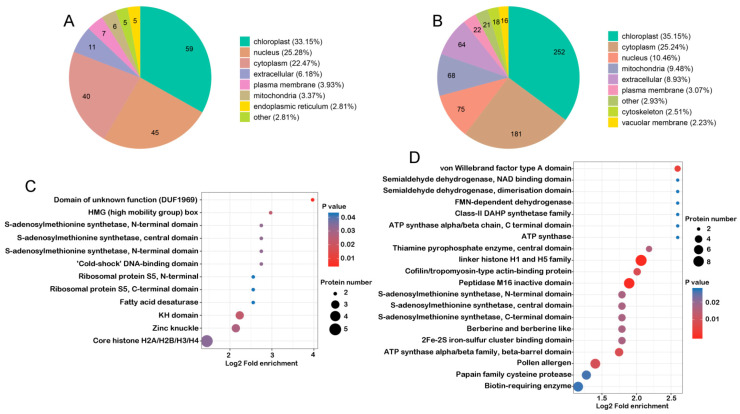
Characteristics of the different comparison groups of anthers. (**A**) Subcellular localization of DEPs in P2 vs. Y2. (**B**) Subcellular localization of DEPs in P4 vs. Y4. (**C**) Enrichment of DEPs domains in P2 vs. Y2. (**D**) Enrichment of DEPs domains in P4 vs. Y4.

To understand the potential function of DEPs, we predicted their subcellular localization in comparison groups P2 vs. Y2 and P4 vs. Y4, respectively. The majority of DEPs identified at stage 2 were assigned to the chloroplast, followed by nucleus and cytoplasm (Figure 5A, Table S4). Similarly, at stage 4, the majority of DEPs were also assigned to the chloroplast, followed by those assigned to the cytoplasm; the number of DEPs assigned to the nucleus, mitochondria, and extracellular matrix was similar but lower than that assigned to the cytoplasm (Figure 5B, Table S4). Compared with stage 2, the proportion of DEPs assigned to the nucleus decreased significantly at stage 4, whereas the proportion of DEPs involved in mitochondria-related functions increased significantly, indicating that these changes in protein distribution in the nucleus and mitochondria potentially have a great impact on plant fertility/sterility. Moreover, DEPs identified in P4 vs. P2 and Y4 vs. Y2 comparisons showed similar subcellular localization patterns (Figure S5A,B, Table S4), i.e., similar proportions of DEPs in the two comparison groups were predicted to be localized to the chloroplast, cytoplasm, nucleus, mitochondria, and extracellular matrix. Interestingly, mitochondria accounted for a large proportion of DEPs in both comparison groups, strongly implying that mitochondria play critical roles in anther development.

To further investigate the functions of DEPs, we performed domain enrichment analysis of DEPs. A total of 12 domains were enriched in DEPs at stage 2, while 20 domains were enriched in DEPs at stage 4 (Figure 5C,D). The C-terminal, central, and N-terminal domains of S-adenosylmethionine (SAM) synthetase were enriched in DEPs at both stages, fatty acid desaturase, ribosomal protein S5, core histone H2A/H2B/H3/H4, and cold-shock DNA-binding domain at stage 2 (Figure 5C), and in linker histone H1 and H5 family, FAD-dependent oxidoreductase, cathepsin propeptide inhibitor domain (I29), NAD-binding domain, and galactokinase galactose-binding signature at stage 4 (Figure 5D). These results are consistent with the results of subcellular localization. Furthermore, analysis of DEPs identified in P4 vs. P2 and Y4 vs. Y2 comparisons revealed the enrichment of 25 and 20 domains, respectively (Figure S5C,D). DEPs harboring these domains were mainly involved in histones, carbohydrate metabolism, energy metabolism, and stress response proteins. All these domain family members affect different processes during anther development [28].

### 2.6. GO Functional Classification and Enrichment Analysis of DEPs in Anthers

**Figure 6:**
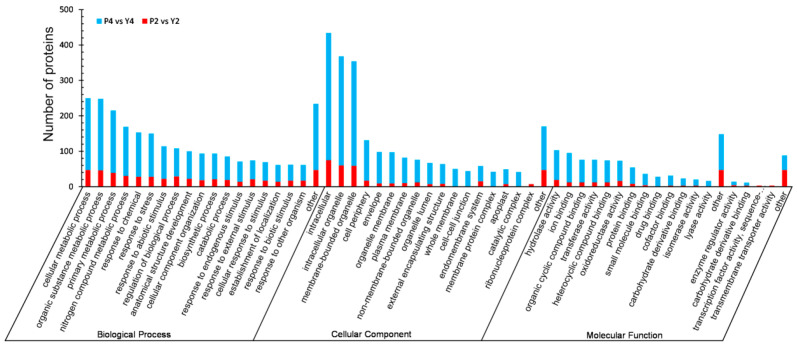
Statistical distribution chart of DEPs under each GO category (2nd level). The x-axis represents GO term and the y-axis displays the number of DEPs in each main category.

**Figure 7:**
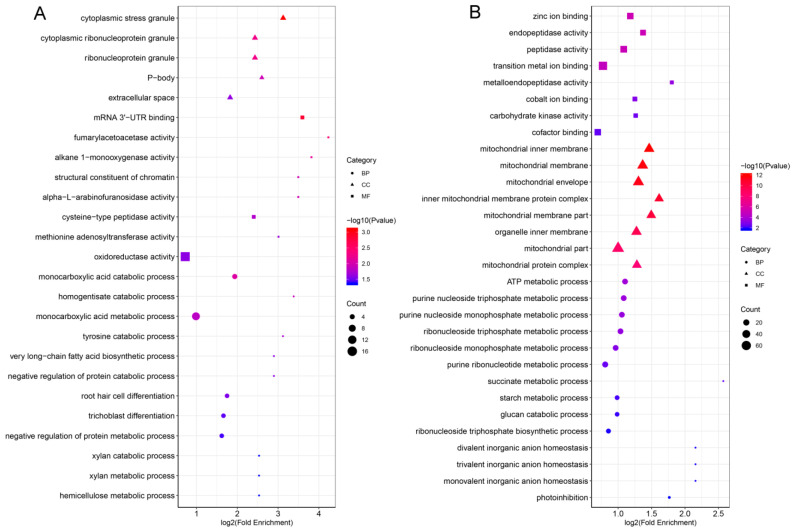
GO (gene ontology) enrichment of DEPs in three categories (BP, biological process; CC, cellular component; MF, molecular function). (**A**) GO enrichment of the comparison group P2 vs. Y2. (**B**) GO enrichment of the comparison group P4 vs. Y4.

To better understand the potential functions of DEPs at the different developmental stages of anthers, we analyzed their GO annotations. DEPs were classified into three GO categories: biological process (BP), cellular component (CC), and molecular function (MF). DEPs of P2 vs. Y2 were grouped into 48 functional categories (19 BP, 14 CC, and 15 MF), while DEPs of P4 vs. Y4 were enriched in 53 functional categories (21 BP, 16 CC, and 16 MF) (Figure 6, Table S5). In the BP category, “cellular metabolic process” and “organic substance metabolic process” were the most enriched terms at the two stages, whereas “cellular component biogenesis” and “developmental process involved in reproduction” were uniquely enriched at stage 4. In the CC category, GO terms including “intracellular”, “intracellular organelle”, and “membrane-bounded organelle” showed the highest enrichment; in addition, “ribonucleoprotein complex”, “photosynthetic membrane”, “cell-cell junction”, and “whole membrane” were uniquely enriched at stages 2 and 4, respectively. In the MF category, DEPs involved in “hydrolase activity”, “organic cyclic compound binding”, “heterocyclic compound binding”, “transferase activity”, “ion binding”, and “oxidoreductase activity” were significantly enriched at the two stages; “transmembrane transporter activity” was uniquely enriched at stage 2, while “lyase activity” and “amide binding” were uniquely enriched at stage 4. Notably, the GO category enrichments were extracted for stages 2 and 4 (Figure 7A,B). In the BP category, “monocarboxylic acid metabolic process” was the main GO term enriched at stage 2, while “ATP metabolic process” and “ribonucleoside triphosphate metabolic process” were the main GO terms enriched at stage 4. In the CC category, “cytoplasmic stress granule” was the main GO term at stage 2, and the GO enrichment terms associated mainly with energy metabolism, including “respiratory chain”, “mitochondrial membrane”, and “mitochondrial inner membrane”, were the main GP terms at stage 4. In the MF category, GO terms associated mainly with enzyme activity, such as “Mrna 3’-UTR binding”, “ubiquitin-protein transferase activity”, and “oxidoreductase”, were enriched at stage 2, whereas “zinc ion binding” and “peptidase activity” were the main GO terms at stage 4. These results indicate clear differences in metabolism at different stages of anther development.

Little difference was observed in the GO functional classification of DEPs between P4 vs. P2 anthers and Y4 vs. Y2 anthers (Figure S6, Table S5). For the CC category, the “lyase activity” GO term was uniquely enriched in the maintainer line. However, GO terms in each comparison were quite different (Figure S7A,B). In the BP category, “DNA geometric change” and “regulation of cellular respiration” were significantly enriched in the P-type CMS line, whereas “starch biosynthetic process” and “acetyl-CoA biosynthetic process” were uniquely enriched in the maintainer line. In the CC category, DEPs involved in dynamic changes in mitochondria were enriched at both stages, with no significant difference. In the MF category, DEPs were mainly involved in energy metabolism and enzyme activity at both stages. These results indicate that energy metabolism, dynamic mitochondrial changes, and enzyme activity are closely related to anther development.

**Figure 8:**
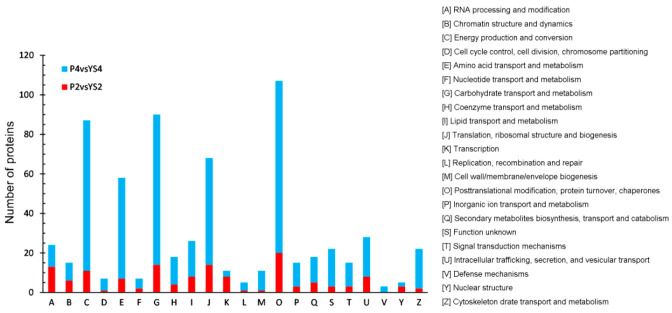
KOG functional classification of DEPs in stage 2 and 4. Capital letters on the x-axis represent the KOG categories listed to the right of the histogram, and the y-axis represents the number of DEPs.

A total of identified DEPs were classified into 21 KOG categories in comparison groups P2 vs. Y2, and 22 KOG in comparison groups P4 vs. Y4, P4 vs. P2, and Y4 vs. Y2 (Figure 8, Figure S8, Table S6). In the P4 vs. P2 comparison, “energy production and conversion” was the largest group (group C; 99 DEPs). In the P2 vs. Y2 comparison, “post-translational modification, protein turnover, chaperones” represented the largest group (group O; 20, 87, and 105 DEPs, respectively), followed by “carbohydrate transport and metabolism” (group G; 14 DEPs) and “translation, ribosomal structure and biogenesis” (group J; 14 DEPs). In the P4 vs. Y4 comparison, “carbohydrate transport and metabolism” (group G; 76 DEPs) and “energy production and conversion” (group G; 76 DEPs) were the largest groups. In the P4 vs. P2 comparison, “energy production and conversion” (group C; 93 DEPs) was the largest group, followed by “translation, ribosomal structure and biogenesis” (group J; 83 DEPs).

### 2.7. KEGG Pathway Enrichment and Cluster Analysis

**Figure 9:**
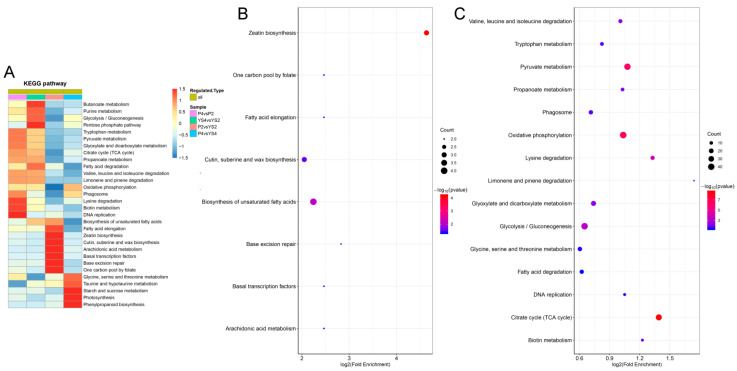
Enrichment analysis of DEPs. (**A**) Heatmap for cluster analysis of the KEGG pathway enrichment. (**B**) KEGG pathway enrichment of the comparison group P2 vs. Y2. (**C**) KEGG pathway enrichment of the comparison group P4 vs. Y4.

To further analyze the biological functions of DEPs, pathway annotation was performed in KAAS. Additionally, statistical significance of KEGG pathway enrichment analysis was determined using two-tailed Fisher’s exact test. In total, 86 and 379 were mapped in eight and nine KEGG pathways at stages 2 and 4, respectively (Figure 9B,C). The “zeatin biosynthesis” and “biosynthesis of unsaturated fatty acids” pathways were mainly enriched at stage 2, whereas “oxidative phosphorylation”, “starch and sucrose metabolism”, and “glycolysis/gluconeogenesis” were the most enriched pathways at stage 4. Additionally, 426 DEPs identified in the P4 vs. P2 comparison were assigned to 16 KEGG pathways, while 432 DEPs identified in the Y4 vs. Y2 comparison were assigned to 14 KEGG pathways (Figure S9, Table S7). Pathways including “citrate cycle (TCA cycle)”, “oxidative phosphorylation”, “pyruvate metabolism”, and “glycolysis/gluconeogenesis” were enriched in both P4 vs. P2 and Y4 vs. Y2 comparisons. In addition, “ribosome” and “lysine” were also enriched in the P4 vs. P2 comparison.

To determine the functional correlation between DEPs in different comparison groups, we conducted cluster analysis of KEGG pathways (Figure 9A). Pathways such as “lysine degradation”, “biotin”, and “DAN replication” were significantly enriched in the P4 vs. P2 comparison, whereas pathways involved in energy metabolism, including “purine metabolism”, “butanoate metabolism”, and “glycolysis/gluconeogenesis”, were mainly enriched in the Y4 vs. Y2 comparison. Additionally, “zeatin biosynthesis”, “basal transcription factors”, and “cutin, suberine, and wax biosynthesis” were mainly enriched at stage 2, whereas pathways involved in energy metabolism, including “phenylpropanoid biosynthesis”, “photosynthesis”, and “starch and sucrose metabolism”, were mainly enriched at stage 4. Thus, the specific pathways of different comparison groups may be the key factors affecting the fertility of anther development and provide important clues for further research on male sterility in wheat.

### 2.8. PPI Networks and Metabolic Pathway Analysis 

**Figure 10:**
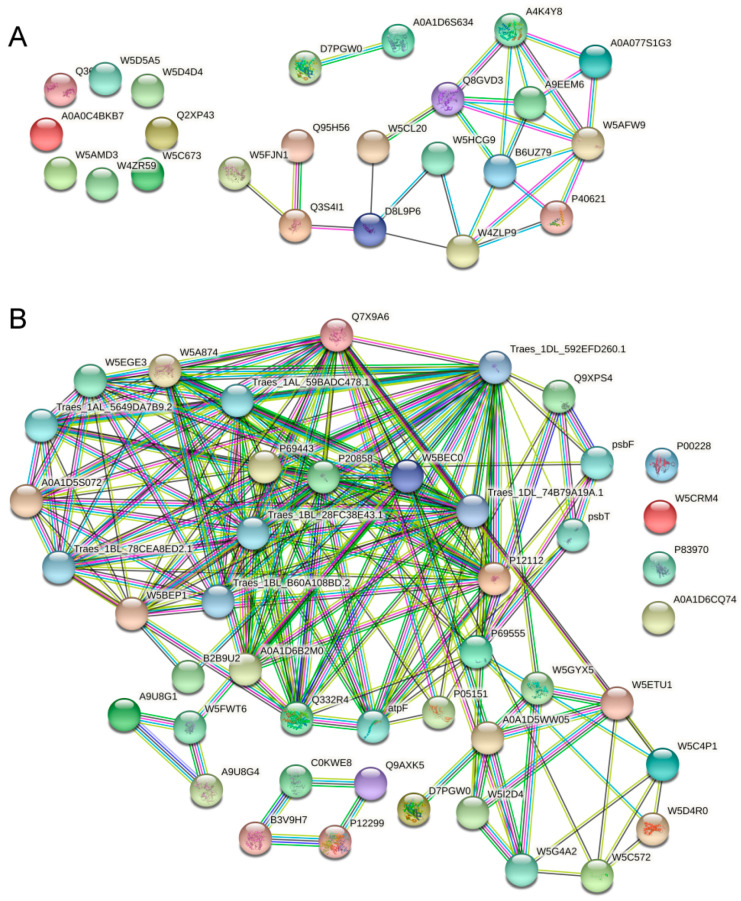
Protein–protein interaction network for DEPs. (**A**) P2 vs. Y2. (**B**) P4 vs. Y4 (which is involved in energy metabolism and carbohydrate metabolism). The network was constructed using the String program (the STRING resource was available online at http://www.string-db.org/) with a confidence score higher than 0.4. Nodes represent proteins, and the line thickness represents the strength of the supporting data.

We searched for proteins differentially expressed between anthers at stage 2 and those at stage 4 using the String 11.0 database, with a confidence score > 0.4 for PPIs. A regulatory network was constructed for DEPs at stage 2 (Figure 10A). In this network, the highest number of nodes was observed for W5AFW9 (connected with seven proteins including A4K4Y8, Q8GVD3, B6UZ79, W4ZLP9, P40621, A9EEM6, and A0A077S1G3), followed by B6U79 (connected with six proteins), A4K4Y8, Q8GVD3, and W4ZLP9 (each connected with five proteins). The second group was constituted by W4ZLP9, W5HCG9, and D8L9P6, which are involved in cellular protein metabolic processes. In addition, three protein pairs (including W5FJN1–Q3S4I1, Q95H56–Q3S4I1, and D7PGW0–A0A1D6S634) were observed (Figure 10A). A previous study showed that photosynthesis, oxidative phosphorylation, starch and sucrose metabolism, TCA cycle, and glycolysis/gluconeogenesis are closely related to plant male sterility. Therefore, we used 153 of those DEPs to generate a PPI network at stage 4. The resulting network was large and complex (Figure 10B), and only one protein pair was observed. Traes_1DL_592EFD260.1 was the most important node (connected with 26 proteins) in this network, followed by Q7X9A26 (connected with 23 proteins) and P69443 (connected with 19 proteins). These results indicate that anther development is regulated by a complex network, and alterations in any of the proteins involved in anther development will result in anther abortion.

### 2.9. Expression Analysis of Genes and Their Cognate DEPs

**Figure 11:**
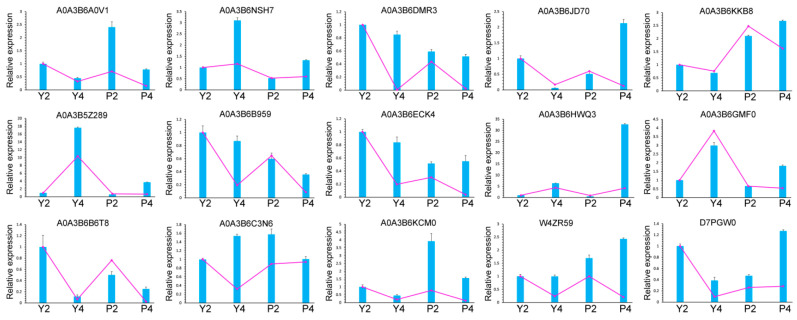
Cognate gene relative expression analysis of DEPs by qRT-PCR. Histograms show protein expression level, whereas line charts show gene relative expression level. The data obtained on genes’ relative expression levels and proteins’ expression levels are the means based on three biological replicates. Y2, Y4, P2, and P4 represent anthers in stage 2, 4, respectively.

To further demonstrate the reliability of proteomic analysis and evaluate the correlation between mRNA and protein expression levels, we performed qRT-PCR analysis. Genes encoding 10 DEPs were randomly selected. The expression profiles of all 15 genes were similar to those of their cognate proteins (Figure 11). Calreticulin (A0A3B6B959), non-specific lipid-transfer protein (A0A3B6B6T8), phytocyanin domain-containing protein (A0A3B6HWQ3), Reticulon-like protein (A0A3B6A0V1), S-adenosylmethionine synthase (A0A3B6NSH7), Fn3_like domain-containing protein (A0A3B6DMR3), Phosphotransferase (A0A3B5Z289), Cinnamyl alcohol dehydrogenase (D7PGW03), and histone H2B (A0A3B6KCM0) proteins and their cognate genes showed exactly the same expression patterns in Y and P anthers, respectively. However, Mitochondrial fission 1 protein (W4ZR59) was upregulated in Y and P anthers, whereas its cognate gene was downregulated; SEC7 domain-containing protein (A0A3B6C3N6) and its cognate gene also showed the completely opposite expression pattern in Y and P anthers. By contrast, the other proteins and their cognate genes showed the same expression pattern in Y and the opposite expression pattern in P-type anthers, respectively. These inconsistencies between protein and transcript levels could be caused by translational or post-translational modifications of the mature protein.

## 3. Discussion

### 3.1. The Pollen Abortion Type of P-Type CMS Line Belongs to Binucleate Microspore Abortion

Many studies have shown that male sterility is caused by various factors and occurs at different developmental stages [29]. Pollen abortion is key for inducing male sterility and can be classified into four types: pollen-free, uninucleate abortive, binucleate abortive, and trinucleate abortive [30]. In our study, microspores were normal, and the tapetum degraded in Y2 and P2. Microspores collapsed, and severe plasmolysis occurred in the late uninucleate stage, but the nucleus was normal (Figure S1E,F). The nuclei and cytoplasm of microspores in P anthers were abnormal, and apoptosis occurred at the binucleate stage (Figure 2G,H and Figure S1G,H). In this stage, compared with its maintainer line, only a few microspores can develop into two nuclei, and the nuclei were diffuse, and cytoplasmic fillers were scarce. These data suggest that the P-type CMS line belongs to binucleate microspore abortion. Further research is needed to gain an in-depth understanding of the processes affecting male sterility. 

### 3.2. Early Tapetum Degradation and Anther Cuticle Defects Are Associated with Male Sterility

Previous studies indicated that abnormal tapetum degeneration plays pivotal roles in pollen abortion in male sterile lines [31,32]. In this study, the analysis of transverse sections of anthers revealed that differences in the development of the tapetal layer occurred earlier in P-type anthers than in Y anthers. At the tetrad stage, the tapetum was dispersed, with obvious vacuolization in P-type anthers (Figure S2). At stage 2, while tapetum degradation was beginning in Y anthers (Figure 3), most tapetum cells were degenerated in P-type anthers (Figure 3). This might lead to the inability of sporogenous cells to absorb sufficient nutrients during the later stages of development and was implicated in binucleate abortive; subsequently, it might have caused aberrant development of pollen grains. Abnormal degeneration of the tapetum at the uninucleate pollen stage causes pollen abortion, as shown previously [33]; similar results were obtained in the present study. Based on these results, we speculate that the expression or function of some genes, proteins, and metabolites involved in tapetum development have undergone significant changes in the P-type CMS wheat line.

In the study of the male sterile mutant, development of anther cuticle is crucial for plant fertility, as has been reported [34]. Wax, Cutin, and two lipophilic biopolymers composed the primary anther cuticle [35]. C16 and C18 fatty acids were directly esterified to glycerol or to each other to form Cutin, finally; and wax is mainly formed from long-chain fatty acids [36,37]. Comparative proteomic analysis of P and Y anthers at stage 2 revealed three DEPs involved in the cutin, suberine, and wax biosynthesis pathway (map00073), which might regulate the development of anther cuticles. Among these three DEPs, A0A3B6JME5 (K20495) and A0A3B6HN30 (K20495), defined as long-chain fatty acid omega-monooxygenases mainly involved in cutin and suberine biosynthesis, and A0A3B6PM30 (K15404), defined as an aldehyde decarboxylase mainly involved in wax biosynthesis, were upregulated in P anthers compared with Y anthers (Figure S10). These results suggest that these three DEPs regulate the contents of cutin and wax to affect anther cuticle development. In addition, A0A3B6EK57 (K18660), A0A3B6N0C3 (K00059), A0A3B6DD50 (K03921), and A0A3B6KFC9 (K03921), involved in fatty acid biosynthesis (map00061), were defined as long-chain-fatty-acid-CoA ligase-like protein, 3-oxoacyl-[acyl-carrier protein] reductase, and acyl-[acyl-carrier-protein] desaturase, respectively. Among these four proteins, A0A3B6N0C3 (K00059) was significantly upregulated, while others were significantly downregulated in P anthers compared with Y anthers (Figure S11). These results are consistent with changes in the contents of cutin and wax and provide further support for anther cuticle development. The cuticle covers the epidermis of the anther and plays a protective role during anther development [38]. The production of anther cuticles depends largely on the timely degradation of tapetum. Early or late degeneration of the tapetum leads to male sterility and defective anther cuticles [39,40]. Our research suggests a close relationship concerning male sterility of P-type wheat between tapetum and anther cuticle.

### 3.3. Starch and Sucrose Metabolism Is a Major Factor Causing Male Sterility in P-Type Wheat

Starch is essential for plant growth and development. Sucrose acts both as an energy source for plants and as a signaling molecule during plant development [41]. In the early development of anthers, starch hydrolysis provides energy for tapetum cell and microspore development, thus resulting in little starch accumulation [42]. Large amounts of starch and polysaccharides accumulate in pollen grains, which facilitates pollen germination; therefore, starch content is closely associated with pollen fertility [43]. All pollen grains of the maintainer line were stained completely black with KI-I_2,_ whereas P pollen grains were stained brown (Figure 2K,L). This indicates that low starch accumulation and male sterility are associated with the absence of starch in pollen grains. However, the reason that no starch accumulates in the male sterile line remains unknown.

**Figure 12:**
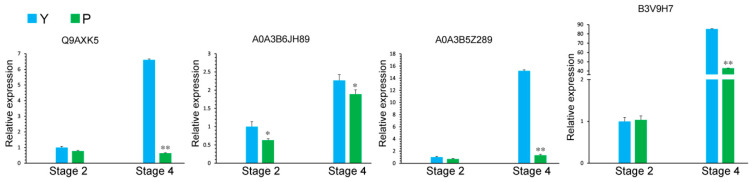
Relative expression of related genes sucrose synthase (A0A3B6JH89), sucrose-6F-phosphate phosphohydrolase SPP2 (Q9AXK5), Phosphotransferase (A0A3B5Z289), and starch synthase (B3V9H7) during anther development in starch and sucrose metabolism. Y (maintain line), P (sterile line). Stage 2: Early uninucleate stage; Stage 4: Binucleate stage. The data were statistically analyzed by Student’s *t*-test (* *p* < 0.05; ** *p* < 0.01).

KEGG enrichment analysis of proteins differentially expressed between P and Y anthers at stage 4 revealed 34 DEPs (Table S8) involved in the starch and sucrose metabolic pathway (Figure 9). This result was consistent with the visual observation of microspore development (Figure S1). Among these DEPs, 24 enzymes were involved in the starch and sucrose metabolic pathway; sucrose synthase (A0A3B6JH89) and phosphotransferase (A0A3B6EJ19) were only slightly upregulated, while Alpha-1,4 glucan phosphorylase (A0A3B6H1W1), sucrose-6F-phosphate phosphohydrolase SPP2 (Q9AXK5), starch synthase, chloroplastic/amyloplastic (B3V9H7), and other related enzymes were significantly downregulated. qRT-PCR was carried out in related genes of sucrose synthase (A0A3B6JH89), sucrose-6F-phosphate phosphohydrolase SPP2 (Q9AXK5), Phosphotransferase (A0A3B5Z289), and starch synthase (B3V9H7) (Table S9). Compared with the maintainer line, the decrease in Q9AXK5 was 0.097-fold, A0A3B6JH89 was 0.835-fold, A0A3B5Z289 was 0.088-fold, and B3V9H7 was 0.504-fold in stage 4 (Figure 12). Our data suggest that the starch and sucrose metabolic pathway was severely affected, leading to little or no starch accumulation in the male sterile anthers from the early uninucleate stage to the binucleate stage of microspores. Further verification of related proteins will be carried out in a future molecular mechanism study. Therefore, we assume that anther development from the early uninucleate stage to the binucleate stage is critical for the induction of fertility in P male sterile wheat.

### 3.4. Carbohydrate and Energy Metabolism

In flowering plants, carbohydrate and energy metabolism is an indispensable and a basic metabolic pathway. The main physiological function of carbohydrate and energy metabolism is to provide energy and carbon sources as well as signaling molecules for the reproductive development of plants [44,45]. Moreover, dysfunction in carbohydrate and energy metabolism can lead to abnormal starch storage in the endothecium and can cause other symptoms, such as tapetal hypertrophy and adverse formation of microspore wall [46]. In the current study, a large proportion of the DEPs were enriched in oxidative phosphorylation (42 DEPs), TCA cycle (24 DEPs), and glycolysis/gluconeogenesis (39 DEPs) pathways (Figure 9, Table S7), and most of these DEPs were downregulated. Additionally, pyruvate dehydrogenase (PDH) E1 component subunits alpha and beta, A0A3B6MUW2, A0A3B6NUN7, and A0A3B6TJ01 were significantly downregulated in the glycolysis/gluconeogenesis pathway in P anthers compared with maintainer line anthers at stage 4, which might have led to inhibition of glycolysis and a decrease in acetyl-CoA; meanwhile, A0A3B6NUN7, A0A3B6MUW2, and A0A3B6TJ01 as subunits of PDH (pyruvate dehydrogenase E1 component subunits alpha and beta) showed the same results. As previously reported, the PDH complex is related to glycolysis/gluconeogenesis, pyruvate metabolism, and the TCA cycle [47]. The TCA cycle plays an important role in the inhibition of PDH activity, leading to anther tapetum swelling or abnormal vacuolation in tobacco plants [48]. Together, these findings suggest the possibility that A0A3B6NUN7, A0A3B6MUW2, and A0A3B6TJ0 are involved in the development of tapetal cells in P-type wheat.

Mitochondria are not only the powerhouse of the cell but also one of the sources of cellular reactive oxygen species (ROS) [49]. Because energy metabolism occurs in mitochondria, defects in mitochondria inevitably affect metabolic processes such as the TCA cycle, respiratory electron transfer, and ATP synthesis [50]. Furthermore, the mitochondrial electron transport chain likely plays an important role in male sterility, as the TCA cycle supplies NADH and FADH2 to the mitochondrial electron transport chain. NADH dehydrogenase, the first enzyme in the mitochondrial electron transport chain, catalyzes the transfer of electrons from NADH to downstream complexes. Previous studies showed that disruption of electron transfer leads to the transfer of excess electrons to molecular oxygen, resulting in the production of ROS [51]. Previous studies suggest that ROS play pivotal roles in induce programmed cell death (PCD) during tapetum degeneration [32,52,53]. In our study, NADH dehydrogenase (A0A3B5Z1D4) and its subunits (A0A3B5ZVN8, A0A1D6CQ74, and A0A3B6C4D6) were downregulated in P-type anthers compared with maintainer line anthers, suggesting reduced electron transport. The slowing down of electron transfer possibly led to the excess ROS accumulation, thus disturbing the redox homeostasis and ultimately inducing premature tapetum degradation. Therefore, disruption of carbohydrate and energy supply is potentially a key factor that affects the normal development of anther tapetum, leading to pollen sterility in P-type CMS wheat.

## 4. Materials and Methods

### 4.1. Plant Material and Anther Collection

A P-type (*primepi*) CMS line of wheat (P; consecutively backcrossed with Yanshi 9 hao) and its maintainer line (Yanshi 9 hao; Y) were used in this study. Growth conditions were exactly the same as other varieties of wheat. In October 2018, plants were grown under natural conditions in an experimental field at Northwest Agriculture and Forestry University, Yangling, Shaanxi Province, P.R. China (108° E, 34° N). In April 2019, anthers of P and Y were collected at the early uninucleate stage (stage 2) and binucleate stage (stage 4) in triplicate, as described previously [54,55], and used for cytological and proteomic analysis. All samples were frozen in liquid nitrogen and stored at −80 °C for further analysis.

### 4.2. Phenotypic Characterization and Microspore Analysis of Anthers

Anthers were visualized under a Motic K400 dissecting microscope (Preiser Scientific, Louisville, KY, USA) and photographed using a Nikon E995 digital camera (Nikon, Tokyo, Japan). Different stages of micropore development were identified by staining the anthers with 1% acetocarmine and 2% iodine–potassium iodide (I_2_–KI). Samples were photographed using a DS-U2 high-resolution camera mounted on a Nikon ECLIPSE E600 fluorescence microscope (Nikon, ECLIPSE, E600, Tokyo, Japan) and analyzed with the NIS-Elements software (Nikon, Tokyo, Japan). Chromosomes were observed by staining with 4′,6-diamidino-2-phenylindole (DAPI; Sigma-Aldrich, Oakville, ON, Canada).

### 4.3. Histological Analysis

Anthers at different developmental stages were fixed in FAA (50% ethanol, 10% formalin, and 5% acetic acid) and dehydrated using an ethanol gradient. The dehydrated anthers were infiltrated with xylene and embedded in Paraplast Plus. Transverse 7-μm-thick sections were placed onto poly-L-lysine-coated slides (Sigma-Aldrich, Darmstadt, Germany) and stained with safranin O/fast green [20]. All images were acquired using the DS-U2 high resolution camera mounted on a microscope (Nikon, ECLIPSE, E600, Tokyo, Japan) and processed with the NIS-Elements software [20]. To examine the structure of anthers in further detail, transmission electron microscopy (TEM) was performed. Anthers at different developmental stages were prefixed in 2.5% glutaraldehyde and embedded in glue [56]. Ultrathin sections (60 nm) were prepared using the UC6 ultramicrotome (Leica, Wetzlar, Germany) and double-stained with 2% (w/v) aqueous uranyl acetate and 2.6% (w/v) aqueous lead citrate. Finally, images were acquired using a HT7700 transmission electron microscope (Hitachi, Tokyo, Japan).

### 4.4. Protein Extraction and Digestion

Proteins were extracted from anthers at different developmental stages, as described previously [57], with slight modifications. Briefly, each anther sample (~0.1 g) was ground to a fine powder in liquid nitrogen and extracted with acetone containing 10% (w/v) trichloroacetic acid (TCA) and 1% (w/v) dithiothreitol (DTT). Samples were stored at −20 °C for at least 4 h and then centrifuged at 4000× *g* for 40 min at 4 °C. The protein pellets were washed three times using pre-cooled acetone and then air-dried in a well-ventilated location. The lyophilized protein powder (~0.03 g) was dissolved in SDT lysis buffer (4% sodium dodecyl sulfate (SDS), 100 mM Tris-HCl, 1 mM DTT (pH 7.6)) and vortexed. The protein pellets were suspended again at 95 °C for 5 min, sonicated 10 times (each 10-s sonication was followed by a 15-s rest period) on ice at 80 W, and then centrifuged (Centrifuge 5810 R, Eppendorf, Hamburg, Germany) at 14,000× *g* for 40 min. The supernatants were collected and filtered through a 0.22-µm filter membrane. Protein in the supernatant was quantified with a BCA Protein Assay Kit (Bio-Rad, Hercules, CA, USA) and stored at −80 °C for subsequent analysis.

Each protein sample (30 µL) was subjected to the filter-aided sample preparation (FASP) procedure, as described previously [58]. Briefly, 200 µL of UA buffer (8 M Urea, 150 mM Tris–HCl (pH 8.0)) was used to remove the detergent (DTT) and other low-molecular-weight compounds and ultrafiltration was repeated (Microcon units, 10 kD). The samples were centrifuged at 14,000× *g* for 15 min. Then, 100 μL of 0.05 M iodoacetamide in UA buffer was added to the protein samples to block reduced cysteine residues, and the samples were incubated in the dark for 30 min. The filter was washed three times with 100 μL of UA buffer and then twice with 100 μL of 25 mM ammonium bicarbonate. Finally, the protein suspension was digested with 3 μg of trypsin (Promega, USA) in 40 μL of 25 mM ammonium bicarbonate overnight at 37 °C, and the resulting peptides were collected as a filtrate. The peptide content was estimated by ultraviolet (UV) light spectral density at 280 nm using a 0.1% (w/v) solution with an extinction coefficient of 1.1, which was calculated on the basis of the frequency of tryptophan and tyrosine residues in vertebrate proteins.

### 4.5. Identification of Peptides

Four groups were provided in biological triplicate for mass spectrometry (MS) analysis. Each sample was desalted using an Acclaim PepMap100 nanoViper C18 high-pressure liquid chromatography (HPLC) column (100 μm × 2 cm; Thermo Scientific, Waltham, MA, USA) and separated on an EASY-nLC 1000 system (Thermo Fisher Scientific, Waltham, MA, USA) using a Thermo Scientific EASY-Column (C18-A2; 10 cm, 75 μm i.d., 3 μm). Samples were eluted using mobile phase A (0.1% formic acid in 2% acetonitrile) and mobile phase B (0.1% formic acid in 84% acetonitrile) over 120 min at a flow rate of 300 nL/min. The MS data were acquired using a data-dependent top 10 method and by dynamically selecting the most abundant precursor ions from the survey scan (300–1800 m/z) for higher-energy C-trap dissociation (HCD) fragmentation. Automatic gain control target was set at 1e6. The dynamic exclusion duration was 60 s. Survey scans were acquired with a 70,000 resolution at m/z 200, and the HCD spectra resolution was set at 17,500 at m/z 200. The normalized collision energy was 30 eV, and the underfill ratio was 0.1%. The instrument was operated in the peptide recognition mode.

The MS data were analyzed using the MaxQuant software [59] (version 1.5.3.17) and searched against the UniProt Triticun_aestivum database in UniProtKB (211,388 total entries, downloaded on 5/14/20). In the initial search, the precursor mass window was set at 6 ppm. The search followed the enzymatic cleavage rule for trypsin: a maximum of two missed cleavage sites, and a 20-ppm mass tolerance for fragment ions. Cysteine carbamidomethylation was considered a fixed modification, while N-terminal acetylation and methionine oxidation of proteins were considered variable modifications for database searches. The cutoff value of the global false discovery rate (FDR) in peptide and protein identification was set at 0.01. The peptides shared between proteins were combined and reported as one protein group [60]. Label-free quantification was performed using MaxQuant (version 1.5.3.17; Protein profiling tools; Germany), as described previously [61]. Intensity-based absolute quantification (iBAQ) was performed in MaxQuant to quantify protein abundance for the identified peptides. An FDR estimation for differentially expressed proteins (DEPs) was performed using a mixture-model-based method [62]. The significance of proteins differentially expressed between samples was examined using the cutoff values of *p* ≤ 0.05 and FDR ≤ 0.05.

### 4.6. Bioinformatics Analysis

DEPs showing a 1.5-fold or greater change in expression and a minimum of two common peptides in all three replicates were analyzed further. First, gene ontology (GO) annotation proteome was derived from the UniProt-GOA database (the database was available online at http://www.ebi.ac.uk/GOA/). Proteins not annotated in the UniProt-GOA database were annotated using the InterProScan software, based on amino acid sequence alignments, to determine their GO functional properties. Then, proteins were classified intro three GO categories: biological process, cellular component, and molecular function. Proteins in each category were analyzed by two-tailed Fisher’s exact test to test the enrichment of DEPs against all identified proteins (*p* ≤ 0.05). The InterPro domain database (the database was available online at http://www.ebi.ac.uk/interpro/) was used to annotate the domains of identified proteins, and two-tailed Fisher’s exact test was employed to test the enrichment of DEPs against all identified proteins (*p* ≤ 0.05). The Kyoto Encyclopedia of Genes and Genomes (KEGG) database was used to annotate the protein pathway, and two-tailed Fisher’s exact test was performed to test the enrichment of DEPs against all identified proteins (*p* ≤ 0.05). Protein–protein interaction (PPI) analyses were performed by submitting the UniProt accession number or sequence of DEPs to the STRING database (database was available online at http://string-db.org).

### 4.7. Quantitative Real-Time PCR (qRT-PCR) Assay

Frozen samples of Y and P anthers (0.1 g each) at stage 2 and stage 4 were ground into a fine powder in liquid nitrogen, and total RNA was extracted using TRIzol Reagent (TaKaRa, Tokyo, Japan). The isolated total RNA was quantified using a Nanodrop 2000 Spectrophotometer (Thermo Scientific, Waltham, MA, USA). Then, 1 μg of total RNA of each sample was reverse-transcribed using a PrimeScript™ RT Reagent Kit (Takara, Shiga, Japan), according to the manufacturer’s instructions, to generate cDNA. Subsequently, qRT-PCR was performed on a BIORAD CFX96 Real-Time System in a 20-μL reaction under the following conditions: initial denaturation at 95 °C for 10 min, followed by 40 cycles at 95 °C 10 min, 95 °C for 15 s and 60 °C for 1 min. The actin gene was used as an endogenous control for normalizing gene expression levels. Primers used for qRT-PCR were designed using Primer Premier 5.0 software and are listed in Table S9. Relative gene expression was calculated using the 2^−ΔΔCt^ method, and each experiment described above was performed in three independent replicates.

## 5. Conclusions

In the present study, we analyzed for the first time the morphology and proteome of anthers of the P-type CMS wheat and its maintainer line Y. Cytological observations indicated that the tapetum degraded earlier in P-type wheat, resulting in the lack of nutrient supply to microspores and preventing their development into normal binucleate cells, thus leading to pollen abortion. Moreover, we identified novel proteins involved in male sterility in wheat. A total of 2576 proteins were quantified in P-type and maintainer anthers. Functional annotation showed that these proteins were significantly enriched in oxidative phosphorylation, glycolysis/gluconeogenesis, TCA cycle, starch and sucrose metabolism, phenylpropanoid biosynthesis, pyruvate metabolism, and other pathways. Based on these results, we concluded that P-type male sterility in wheat is likely caused by the disruption of carbohydrate metabolism, energy supply, and protein synthesis. Overall, these results elucidate the pollen abortion type of P-type CMS line and several important metabolic pathways that are involved in anther development. They provide directions for further research on P-type sterility mechanisms and serve as a valuable resource for researchers in plant biology, in general, and plant sexual reproduction, in particular.

## Data Availability

The data presented in this study are available in the article and supplementary material.

## Supplementary Materials

The following are available online at https://www.mdpi.com/1422-0067/22/4/2012/s1.
